# Mental health of fishermen: current research status and future directions—insights from seafarer studies (a scoping review)

**DOI:** 10.3389/fpubh.2026.1783401

**Published:** 2026-06-22

**Authors:** Wenhao Lin, Yu Fang, Haidong Song

**Affiliations:** 1 Ruian Fifth People's Hospital, Wenzhou, China; 2 Affiliated Mental Health Center & Hangzhou Seventh People's Hospital, Zhejiang University School of Medicine, Hangzhou, China; 3 Mental Health Center Zhejiang University School of Medicine (Hangzhou Seventh People's Hospital), Hangzhou, China

**Keywords:** fishermen, influencing factors, interventions, manifestations, mental health, occupational health, scoping review, seafarers

## Abstract

**Background and objective:**

This scoping review systematically mapped current research on fishermen's mental health. Using the more methodologically mature field of seafarer research as a reference, it aimed to identify key limitations and future directions for the fishermen's mental health domain.

**Methods:**

Guided by the PRISMA-ScR framework, a systematic search of Chinese and English databases was conducted. Studies on fishermen and seafarers were included, categorized, and comparatively analyzed.

**Main findings:**

Analysis of the 41 included studies (This paper cites 46 articles; 41 of which are obtained by the method of review. The additional 5 references are theoretical or methodological articles cited in the introduction and methods sections to provide background and methodological justification) revealed several insights. First, there is a severe shortage of high-quality empirical research on fishermen. Study designs are dominated by cross-sectional surveys, and reports of issues like burnout and sleep disorders show regional variations. Second, factors influencing fishermen's mental health operate at multiple levels. Occupational environmental factors (e.g., high-intensity labor) represent a core shared challenge for both groups. However, fishermen's mental health is also subject to more direct and acute impacts from policy and economic factors (e.g., fishing bans, income instability). It is further moderated by socio-cultural factors (e.g., norms emphasizing resilience that deter help-seeking). Finally, evidence-based psychological interventions for fishermen are extremely scarce. Existing studies are largely confined to theory or policy recommendations. Explorations in the seafaring domain—regarding mental health education, institutionalized rest, and technology-enabled support—provide clear directions for designing interventions tailored to fishermen's working conditions.

**Conclusion and outlook:**

Future research should prioritize large-scale, nationally representative baseline surveys of fishermen's mental health. Building on seafarer research, efforts should focus on constructing an integrated intervention system. This system should encompass livelihood security policies, community support networks, and the application of digital technologies.

## 1 Introduction

Fishermen primarily refer to laborers who live and work long-term at sea, engaging in fishing as their main occupation. Fisheries are a livelihood source for tens of millions globally, yet attention to their importance and the mental health of fishermen remains limited (1). As a high-risk occupation, fishermen face severe chronic stress with significant health impacts, including an increasing risk of suicide (2, 3). Despite extensive academic discussions on biophysical issues such as overfishing (4), it is strikingly rare that attention has been paid to the psychological trauma these issues inflict on fishermen's lives. This paper argues that this humanistic dimension represents a critical yet long-neglected consequence of the current fisheries crisis. Strengthening their conscious psychological identity is significant for promoting ethnic unity and achieving mutual benefits among communities (5). However, current research is fragmented, with evidence largely stemming from localized cross-sectional surveys and a lack of validated, effective intervention programs. A survey in Bangladesh indicated fishing as one of the most unstable occupations with high rates of threats and hazards (6). Local surveys in Chinese coastal cities found that fishermen face severe livelihood sustainability challenges after fishing bans, with uncertainty about the future fostering negative psychological states such as anxiety and depression (7, 8). In contrast, research on the mental health of seafarers—a comparable group of maritime workers—has accumulated relatively mature theoretical and practical experience. Studies have found that mental health status directly or indirectly influences seafarers' life satisfaction (9). Furthermore, a study by Ugurlu O et al. indicates that mental health issues increase accident risks by approximately 2.5 times compared to physical health problems. Factors such as stress, occupational burnout, and mental fatigue significantly impair decision-making capacity, situational awareness, and communication efficiency. In contrast, physical fatigue caused by prolonged working hours, shift-based operational schedules, and sleep deprivation has a relatively limited impact. The research demonstrates that physical measures alone are insufficient to ensure maritime safety; comprehensive measures encompassing psychological support, workload management, and mental health assessments must be implemented (10). Therefore, this scoping review aims to systematically map the evidence landscape of fishermen's mental health and, using seafarer research as a reference frame, identify key gaps through comparison, ultimately providing evidence-based insights for developing a localized mental health promotion system for fishermen.

## 2 Methods

### 2.1 Methodology introduction

This study selects scope reviews as the most suitable methodological approach. Unlike systematic reviews (which typically address specific clinical questions by integrating high-quality research evidence), scope reviews are better suited for mapping key concepts, types of evidence, and research gaps in emerging fields. A review is an evidence synthesis aimed at identifying and mapping relevant evidence that meets predefined inclusion criteria, involving the reviewed subject, field, context, concept, or issue. The review questions guiding scope reviews are generally broader than those of traditional systematic reviews. Scope reviews may include various types of evidence (e.g., different research methods, primary studies, reviews, non-empirical evidence). Since scope reviews aim to provide a comprehensive overview of evidence rather than quantitative or qualitative synthesis of data, methodological evaluation or bias risk assessment of the sources included in scope reviews is usually not required. Scope analysis reviews systematically identify and map relevant literature that meets predetermined inclusion criteria to address specific objectives and review questions related to key concepts, theories, data, and evidence gaps (11). This approach allows us to systematically examine the extent, range, and nature of research activity on fishermen's mental health, identify gaps, and use the seafarer literature as a comparative lens, without being restricted by study design. This method has been increasingly used in public health and occupational health research over the past decade to provide a landscape overview of complex issues. For example, Heming M's survey on the mental health of German farmers (12). This and Dwarika MS's study on mental health in dance (13). This both employed this method.

### 2.2 Study design

This study is a scoping review conducted following the PRISMA-ScR reporting guidelines, designed to systematically map existing evidence in the field of fishermen's mental health and identify research gaps.

### 2.3 Literature search strategy

The search employed a dual-path strategy targeting both the “fishermen” and “sailors” groups to comprehensively cover high-quality literature in these two fields. The detailed search strategy is documented in Table 1.

**Table 1:** Search strategy.

Project	Content
Search time range	2015-01-01 to 2025-12-31
Linguistic constraint	English/Chinese
Document management tools	EndNote
Cbase	CNKI
Chinese search query	SU = (‘渔民'OR‘渔工'OR‘渔业工人'OR‘捕捞从业者'OR‘退捕渔民') AND (‘心理健康'OR‘心理压力'OR‘抑郁'OR‘焦虑'OR‘倦怠'OR‘睡眠障碍'OR‘心理干预'OR‘社会支持') OR SU = (‘海员'OR‘船员'OR‘航海人员') AND (‘心理健康 'OR‘职业压力'OR‘工作压力'OR‘疲劳'OR‘心理适应').
English database	PubMed, Web of Science, Scopus ((fishermen [MeSH Terms] OR fisherman [tiab] OR fisher [tiab] OR “commercial fishing” [tiab] OR “fishing” [tiab]) “industry” [tiab]) OR (seafarers [MeSH Terms] OR seafarer [tiab] OR mariner [tiab] OR “merchant navy” [tiab] OR “ship crew” [tiab])) AND ((“Mental Health” [MeSH] OR “Mental.”
PubMed search query	Disorders “[MeSH] OR” Stress, “Psychological” [MeSH]) OR (mental health [tiab] OR psychological distress [tiab] OR depress [tiab] OR anxiety [tiab] OR stress [tiab] OR burnout [tiab] OR PTSD [tiab] OR intervention^*^[tiab] OR support [tiab])) NOT (animals [mh] NOT humans [mh]).
Web of Science search query	TS = (((fishermen OR fisherman OR fisher OR “commercial fishing” OR “fishing industry”) OR (seafarers OR seafarer OR mariner OR “merchant navy” OR “ship crew”)) AND ((“mental health” OR “psychological distress” OR depress^*^OR anxiet^*^OR stress OR burnout OR PTSD OR intervention^*^OR support))) AND LANGUAGE: (English OR Chinese) AND DOCUMENT TYPES: (Article OR Review) time frame: 2015-01-01 to 2025-12-31 OR Chinese) AND DOCUMENT TYPES: (Article OR Review).
Scopus search query	TITLE-ABS-KEY (((fishermen OR fisherman OR fisher OR “commercial fishing” OR “fishing industry”) OR (seafarers OR seafarer OR mariner OR “merchant navy” OR “ship crew”)) AND ((“mental health” OR “psychological distress” OR depress^*^OR anxiet^*^OR stress OR burnout OR PTSD OR intervention^*^OR support))) AND (LIMIT-TO (PUBYEAR, 2025) OR LIMIT-TO (PUBYEAR, 2024) OR LIMIT-TO (PUBYEAR, 2023) OR LIMIT-TO (PUBYEAR, 2022) OR LIMIT-TO (PUBYEAR, 2021) OR LIMIT-TO (PUBYEAR, 2020) OR LIMIT-TO (PUBYEAR, 2019) OR LIMIT-TO (PUBYEAR, 2018) OR LIMIT-TO (PUBYEAR, 2017) OR LIMIT-TO (PUBYEAR, 2016) OR LIMIT-TO (PUBYEAR, 2015)) AND (LIMIT-TO (LANGUAGE, “English”) OR LIMIT-TO (LANGUAGE, “Chinese”))

**Figure 1:**
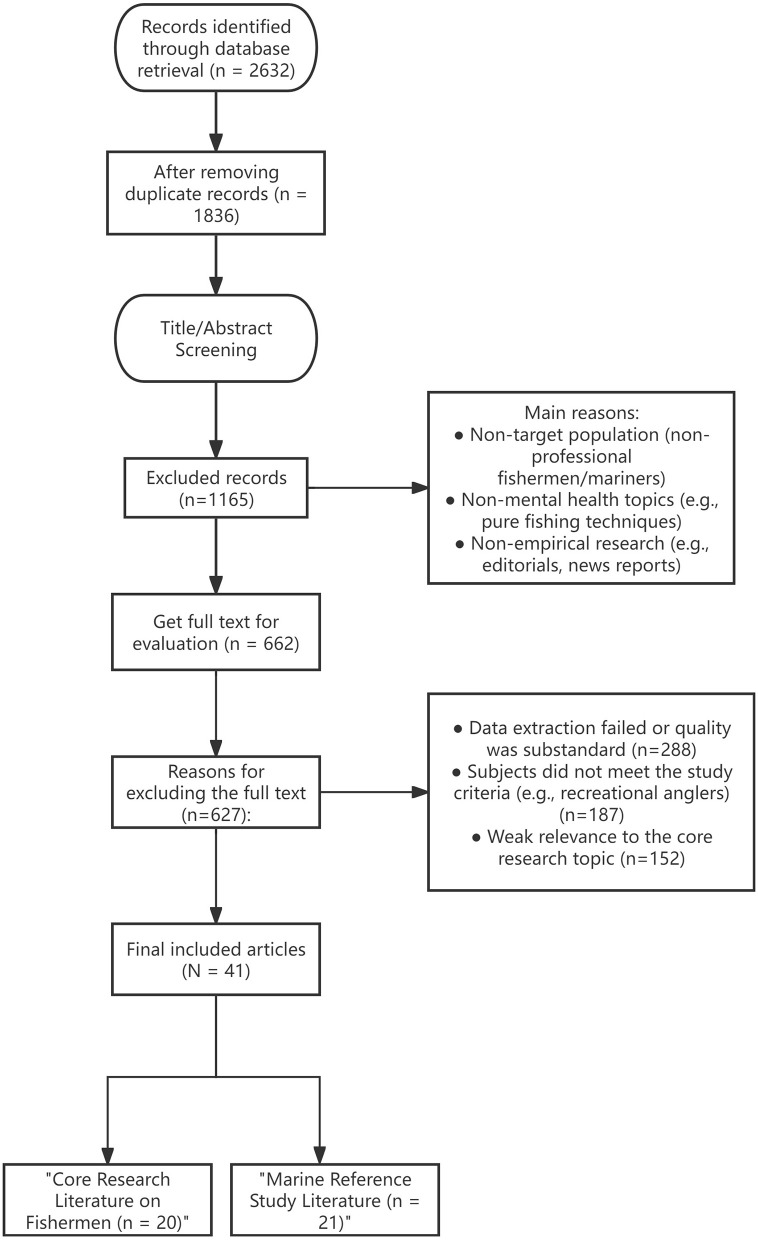
Literature screening process.

The literature screening process is illustrated in Figure 1, resulting in the inclusion of 41 studies.

To ensure transparency, the inclusion and exclusion criteria are detailed in Table 2.

**Table 2:** Literature inclusion and exclusion criteria.

Screening dimension	Inclusion criteria	Exclusion criteria
Study population	Professional fishermen (including commercial, individual) or professional seafarers (merchant, passenger ship crews).	Recreational anglers, naval personnel, inland waterway crews, fish processing workers.
Study content	Empirical studies assessing mental health status, analyzing risk/protective factors, or evaluating interventions effectiveness. High-quality theoretical or review articles providing frameworks were considered.	Studies focusing solely on physical injuries, fishery resource management, etc., without clear mental health variables or in-depth discussion.
Study design	Quantitative (cross-sectional, cohort, experimental), qualitative, and mixed-methods studies.	Conference abstracts without full text, commentaries, news reports.
Publication features	Published between Jan 1, 2015, and Dec 31, 2025; Chinese or English.	Non-Chinese/English publications; full text unavailable.
Quality requirement	Clear description of research methods and extractable data.	Unclear methodology or major methodological flaws.

All seafarer studies meeting the basic PICO criteria (population: professional seafarers; concept: mental health outcomes; context: maritime occupational settings) were included regardless of their explicit relevance to fishermen. During the data extraction and narrative synthesis phase, the research team thematically prioritized findings that offered direct comparability, mechanistic insights, or practical implications for fishermen's mental health. This analytical prioritization is clearly distinguished from *pre-hoc* inclusion/exclusion screening and is described transparently in the synthesis, thus avoiding circularity and confirmation bias.

### 2.4 Data extraction and analysis

A standardized form was used to extract data, including author, country, study design, sample, and key findings. Descriptive statistics and narrative synthesis were employed. The analysis first examined the overall characteristics of fishermen studies. Subsequently, findings from fishermen research were compared with those from sailor studies across three themes—“problem manifestations,” “influencing factors,” and “intervention measures”—to elucidate similarities, differences, and implications.

### 2.5 Study protocol and literature screening process

Since the scope review is currently not accepted for registration by PROSPERO (in accordance with PROSPERO's official registration criteria), this review is not registered with PROSPERO. However, to ensure methodological rigor and transparency, the research team adhered to the PRISMA-ScR guidelines and developed an internal study protocol in advance. This protocol included the following core elements: (1) primary research questions, (2) search strategies (including databases, search terms, and time ranges, see Table 1), (3) study inclusion criteria (see Table 2), (4) data extraction forms (specifying the specific information to be extracted from each study), and (5) data integration plan (i.e., descriptive summaries, and narrative comparisons across the three thematic domains). The protocol provided a roadmap for the review team and guided all subsequent screening and analysis phases.

Literature screening and data extraction were conducted independently by two researchers (Lin WH and Fang Y). After managing and removing duplicates using EndNote, the process involved sequential title/abstract screening and full-text review. Disagreements were resolved through discussion or by arbitration from a third researcher (Song HD). For the inclusion criterion regarding “high-quality theoretical or review articles,” specific criteria were established: the article must be published in a peer-reviewed journal, and (1) theoretical articles should propose a clear conceptual framework or mechanistic model; (2) review articles should employ systematic methods (e.g., systematic review, scoping review) or offer significant integrative contributions to the field. The final inclusion of such articles was subject to collective review by the research team.

## 3 Results

### 3.1 Characteristics of included studies

Among the 41 included studies, 20 directly investigated fishermen's mental health. This body of research exhibits strong regional limitations and remains largely at the stage of descriptive and correlational analysis. The initial comparison highlights the relative underdevelopment of research in the fishermen domain. Detailed findings are recorded in Table 3.

**Table 3:** Comparative characteristics of fishermen's mental health research.

Characteristic dimension	Fishermen studies (*n* = 20)	Seafarer studies (*n* = 21)	Implication of differences
Geographic focus	Highly concentrated in specific regions.	More geographically diverse, including more international studies.	Fishermen's research has a strong regional bias, limiting generalizability; seafarer research has a broader international perspective.
Study design	Overwhelmingly cross-sectional surveys; no RCTs.	Also predominantly cross-sectional but includes mixed-methods, cohort studies, and systematic reviews, showing greater methodological diversity.	Fishermen research is almost entirely descriptive/correlational, lacking causal exploration and intervention evidence.
Theoretical application	Most studies did not explicitly mention or apply psychological theoretical frameworks.	Nearly half explicitly guided by theories (e.g., job demands-resources model, ecological models).	Fishermen's research often lacks theoretical grounding, limiting depth; seafarer research shows greater theoretical awareness.

To substantiate the methodological quality of the included fishermen studies, we performed a quality appraisal using the Joanna Briggs Institute (JBI) Critical Appraisal Checklist for Analytical Cross-Sectional Studies (2020 version). This tool is designed to assess the risk of bias in cross-sectional studies investigating etiological or risk factors, and is widely used in evidence synthesis.

The checklist comprises eight items: (1) clear definition of inclusion criteria; (2) detailed description of study subjects and setting; (3) valid and reliable measurement of exposure; (4) use of objective, standard criteria for condition measurement; (5) identification of confounding factors; (6) stated strategies to deal with confounding; (7) valid and reliable measurement of outcomes; and (8) appropriate statistical analysis. Each item was rated as “yes” (clearly met), “no” (not met), or “unclear” (insufficient information reported). A total score (range: 0–8 points) was calculated for each study. Studies scoring ≥6 points were considered to have a low risk of bias, those scoring 4–5 points had a moderate risk, and those scoring ≤ 3 points had a high risk. Both assessments were conducted independently by two reviewers; any discrepancies were resolved through discussion. Two reviewers (Lin WH and Fang Y) independently assessed each study; disagreements were resolved by discussion or by a third reviewer (Song HD). The full appraisal results are presented in Table 4.

**Table 4:** Quality assessment of 16 cross-sectional fisheries studies using the JBI Cross-sectional study analysis checklist (eight items).

References	Q1	Q2	Q3	Q4	Q5	Q6	Q7	Q8	Score (/8)
Laraqui et al. (2)	✓	✓	✓	✓	χ	χ	✓	✓	6
Alicandro et al. (3)	✓	✓	✓	✓	✓	✓	✓	✓	8
Zakaria et al. (6)	✓	✓	χ	χ	χ	χ	χ	✓	3
Wu et al. (7)	✓	✓	✓	✓	✓	χ	✓	✓	7
Wu et al. (8)	✓	✓	χ	χ	χ	χ	χ	✓	3
Laraqui et al. (14)	✓	✓	✓	✓	χ	χ	✓	✓	6
Laraqui et al. (15)	✓	✓	✓	✓	χ	χ	✓	✓	6
Stoll et al. (19)	✓	✓	χ	χ	χ	χ	χ	✓	3
Jiang et al. (20)	✓	✓	✓	χ	χ	χ	χ	✓	4
Yadav et al. (23)	✓	✓	✓	✓	χ	χ	✓	✓	6
Remmen et al. (24)	✓	✓	✓	χ	χ	χ	χ	✓	4
Miñarro and Selim (25)	✓	✓	✓	χ	✓	χ	✓	✓	6
Chen et al. (33)	✓	✓	✓	✓	✓	χ	✓	✓	7
Liang et al. (34)	✓	✓	χ	χ	χ	χ	χ	✓	3
Xu et al. (41)	✓	✓	✓	χ	χ	χ	✓	✓	5
Putri et al. (44)	✓	✓	✓	χ	✓	χ	✓	✓	6

### 3.2 Manifestations of mental health problems: preliminary sketch and evidence gaps

Fishermen studies have begun to sketch the contours of their mental health issues, but significant gaps in depth and systematization remain compared to seafarer research. Detailed findings are recorded in Table 5.

**Table 5:** Evidence comparison for manifestations of mental health problems.

Problem type	Evidence in fishermen studies	Reference and depth from seafarer studies	Comparison and implication
Occupational burnout	Laraqui et al. (14) on Moroccan fishermen reported specific prevalence (16.1% emotional exhaustion) and identified long working hours (>14 h) as a key risk. This is one of the few quantitative studies.	No epidemiological studies directly assessing burnout prevalence in seafarers was identified in this search.	Fishermen research provides point estimates but lacks cross-cultural, large-sample prevalence data and in-depth mechanistic analysis.
Sleep disorders	Another study by Laraqui et al. (15) reported 47.2% of fishermen with chronic insomnia, linking it to fatigue and socio-economic issues.	Galić et al. (16) not only reported 39% of seafarers having sleep problems, but also used mixed methods to explore the complex relationship between shift systems, onboard environment, and sleep quality.	Both confirm high prevalence. Seafarer research demonstrates how to move beyond prevalence to contextualized, mechanistic inquiry.
PTSD	No epidemiological studies directly assessing PTSD prevalence in fishermen were identified; related discussions are largely theoretical.	Szafran et al. (17) reported that 36% of seafarers experienced traumatic events and nightmares. Lazuk et al. (18) provided high-quality evidence on long-term psychological reactions of maritime disaster survivors.	PTSD risk in high-accident-risk fishermen is severe. Under-researched. Seafarer evidence highlights its importance and potential methodologies.
Addictive behaviors	Addictive behaviors negatively impact their lives and mood. Stoll et al. (19) noted widespread alcohol use among fishermen and seafarers, with 61.0% of drinking fishermen experiencing accidents. Jiang et al. (20) revealed a “work stress → nicotine dependence → depression” mediation path.	No epidemiological studies directly assessing addictive behavior prevalence in seafarers were identified.	Fishermen research contributes to mechanistic exploration of addiction but similarly lacks intervention studies.

### 3.3 Influencing factors: shared occupational core and fishermen's unique vulnerabilities

Synthesized analysis indicates that fishermen's mental health is influenced by multi-level factors. Table 6 summarizes core findings and highlights similarities and differences between evidence for seafarers and fishermen.

**Table 6:** Evidence comparison of influencing factors on mental health for fishermen and seafarers.

Factor level	Specific factors	Evidence in fishermen studies	Evidence in seafarer studies	Key differences & implications
Core occupational environment	1. Physical hazards	Confirmed: occupational noise exposure linked to health issues (21).	Confirmed: seafarers recognize noise hazards but protection is inadequate (22, 23).	Consensus: both confirm as risk factors. Difference: seafarer research focuses more on perception and behavior (PPE use).
	2. Work stress	Confirmed: physical workload significantly correlates with fatigue (24). No close relationship found between actual catch and positive impact (25).	Confirmed: further analyzed via models quantifying work-family conflict, satisfaction, etc. (26–28).	Consensus: high-intensity, long hours are common stressors.
	3. Social isolation	No identified empirical studies	Confirmed, and linked to turnover intention, mood (9, 29–31).	Key gap: risk is identified and quantified in seafarers but entirely absent in fishermen research, pointing to a potentially neglected area.
	4. Interpersonal risk	No identified empirical studies	Confirmed: significant proportion of seafarers experience verbal/physical (32).	Key gap: quantified in seafarer research but absent in fishermen studies, indicating a critical research oversight.
Policy/economic shocks	5. Direct policy impact	Clearly prominent: studies focus on psychological crisis from policies (e.g., fishing bans) causing livelihood disruption, linked to vulnerable demographics (2, 33, 34).	No identified empirical studies	Most distinct difference: macro policies are a direct, severe risk source unique to fishermen, highlighting their structural vulnerability. seafarer research contrasts this.
Socio-cultural moderators	6. Beliefs and help-seeking	Confirmed: “Accepting risk, downplaying harm” culture hinders help-seeking and service access (35).	No identified empirical studies	Contextual difference: fishermen are constrained by traditional community culture, requiring entirely different intervention entry points.

The analysis reveals three interrelated levels of influence. First, occupational environmental factors (e.g., noise, high-intensity labor, social isolation) represent a common core contributing to mental health impairment for both groups, with mutual corroborating evidence. Second, policy and economic factors constitute a unique and potent source of shock for fishermen, such as the direct livelihood and psychological crises triggered by “fishing ban” policies, a concern not mirrored to the same degree in seafarer research, underscoring the structural roots of fishermen's psychological risk. Finally, socio-cultural factors play differing moderating roles: fishermen are deeply influenced by localized ‘resilience' culture and traditional community networks. Notably, empirical evidence regarding risks like workplace violence and social isolation is completely absent in fishermen's studies—a stark contrast to clear findings in seafarer research, indicating a significant research gap.

### 3.4 Intervention measures: scarcity in fishermen's practices and reference from seafarer experience

Current research on psychological interventions for fishermen is extremely scarce, forming a stark contrast with exploratory efforts in the seafaring domain. Detailed findings are recorded in Table 7.

**Table 7:** Intervention measures comparison and evidence-based implications.

Intervention direction	Current state in fishermen's research	Effective explorations and evidence in seafarer research	Implications for fishermen field
Psychoeducation and skill training	No identified empirical studies.	Abila and Acejo (36) emphasized the importance of proactive mental health education. Chen (37) found casework interventions alleviated seafarer psychological fatigue. Le Gac and Texier (38) trained seafarers via simulation to identify distress, with 97% reporting increased confidence, demonstrating feasibility and acceptability. Paranthatta (39) found that meditation can serve as a daily practice for sailors to manage anxiety and improve sleep quality on board.	Direct reference: structured, practical skill training for maritime workers is feasible and necessary, serving as a starting point for fishermen interventions.
Organizational policy and rest systems	Mostly policy recommendations, e.g., appeal considering social life characteristics (40), improving social security (41); lacks evaluation of psychological impacts of existing policies (e.g., fishing moratorium).	Xiao et al. (42) linked social support to seafarer health. An et al. (43) empirically demonstrated that scientific leave schedules significantly correlated with reduced fatigue and better return-to-work states.	Methodological Reference: shift from “making recommendations” to “evaluating existing policy effects,” providing localized evidence for basic protections like working hours and leave.
Communication tech. and social support	Putri et al. (44) found fishermen's internet use correlated with increased subjective wellbeing—a rare positive finding—but not developed into an intervention tool.	Pauksztat et al. (45) confirmed good internet access was a key protective factor for seafarers mental health during pandemic isolation. Abila et al. (46) reviewed trends in mental health apps, hotlines, etc.	Pathway reference: strongly supports improving communication infrastructure and developing digital mental health tools as core strategies to overcome geographic isolation and enhance service accessibility for fishermen.

## 4 Discussion

### 4.1 Summary of key findings

Seafarers are employed by highly organized shipping companies and operate under international maritime regulations and corporate management systems, facilitating systematic sampling and research. In contrast, fishermen typically work within family units, community groups, or informal labor organizations—being dispersed, mobile, and lacking centralized support structures—which significantly hinders research accessibility and data collection. This structural disparity yields three key consequences: first, fishermen are more vulnerable to direct psychological impacts from macro-level policies (e.g., fishing moratoriums) due to the absence of corporate safeguards and union support; Second, their coping behaviors are deeply influenced by traditional community norms (such as resilience culture), whereas seafarers are more affected by organizational structures and occupational health regulations; Third, the prevalent “evidence gaps” identified in fishery studies (e.g., social isolation, workplace violence) reflect methodological challenges rather than the absence of these risks. Consequently, future research should not simply replicate seafarer intervention strategies but must be tailored to the institutional and cultural contexts specific to fishermen.

### 4.2 Recommendations for future research and practice

Based on the identified gaps, future work should prioritize the following.

#### 4.2.1 Strengthen the research foundation

Short-term: conduct targeted, cross-sectional surveys in high-priority areas (e.g., fishing ban zones).

Long-term: secure funding for a nationally representative, longitudinal cohort study.

#### 4.2.2 Initiate “focused” intervention research

Short-term: pilot-test a seafarer-derived psychoeducation module with one fishing cooperative.

Long-term: design and implement a multi-arm rct comparing different intervention strategies (e.g., digital tool vs. community liaison).

#### 4.2.3 Technology-enabled support

Short-term: develop a simple sms-based mental health tip service accessible via satellite phone.

Long-term: collaborate with telecom providers to integrate a comprehensive mental health app into subsidized data plans for fishermen. Community-Embedded Support: short-term: train a small cohort of “peer supporters” at major ports. Long-term: integrate mental health literacy into the standard curriculum for fisheries vocational training.

#### 4.2.4 Policy-linked evaluation

Short-term: add brief, validated mental health scales to existing government surveys of fishermen's livelihoods.

Long-term: advocate for a legislated mandate for mental health impact assessments for all major fisheries policy changes.

### 4.3 Quality assessment

The quality appraisal of 16 cross-sectional fishermen's studies (Table 8) revealed major methodological limitations. The mean JBI score was 5.2/8; only half (8/16) scored ≥6. The most common deficiencies were lack of confounder adjustment (81%), unvalidated measurement tools (38%), and the absence of objective criteria (38%). These weaknesses limit causal inference, cross-study comparability, and the reliability of prevalence estimates. Future studies should prioritize validated scales, control confounders, and standardized diagnostic criteria to strengthen the evidence base.

**Table 8:** List of 41 included studies.

No.	Author(s)	Year	Title	Journal/ source	Key themes/ keywords	References
1	Laraqui O, et al.	2018	Occupational risk perception, stressors, and stress of fishermen.	Int Marit Health	Fishermen, occupational risk perception, stressors, and stress.	(2)
2	Alicandro G, et al.	2021	Mortality from suicide among agricultural, fishery, forestry, and hunting workers in Italy and the contribution of work-related factors.	Occup Environ Med	Fishery workers, suicide mortality, work-related factors.	(3)
3	Deng Q.	2023	Cultivation of Hainan fishermen's conscious psychological identity as a Chinese national community: research on the protection and inheritance of maritime intangible cultural heritage.	Cult Ind	Hainan fishermen, psychological identity, cultural heritage (Chinese).	(5)
4	Zakaria MUMA, et al.	2022	Evaluation of occupational health management status and safety issues of the small-scale fisheries sector in Bangladesh.	Int Marit Health	Small-scale fisheries, occupational health, and safety management.	(6)
5	Wu Z, et al.	2023	Compensation mechanisms for fishermen quitting fishing: a case of Jiangsu province, China.	Heliyon	Retired fishermen, compensation mechanisms, China.	(7)
6	Wu J, et al.	2016	Survey on the Survival Status of Fishermen in Zhejiang Province and Analysis of Countermeasures: a Case Study of Wenzhou, Taizhou, Ningbo, and Zhoushan.	Chinese Journal of Market	Fishermen, survival status, livelihood analysis (Chinese).	(8)
7	Baygi F, et al.	2022	Seafarers' mental health status and life satisfaction: structural equation model.	Front Public Health	Seafarers, mental health, life satisfaction, SEM.	(9)
8	Ugurlu Ö, et al.	2025	Analysis of the Impact of Officers of the Watch's Mental and Physical Health Issues on Collision and Grounding Accidents.	Inquiry	Seafarers, mental/physical health, maritime accidents.	(10)
9	Laraqui O, et al.	2023	Burnout syndrome of coastal fishermen.	Int Marit Health	Coastal fishermen, burnout syndrome.	(14)
10	Laraqui O, et al.	2022	Health status, sleeping habits, and dyssomnia of coastal fishermen.	Int Marit Health	Coastal fishermen, health status, sleep habits, dyssomnia.	(15)
11	Galić M, et al.	2023	“I Constantly Feel Worn Out”: mixed-methodology Approach to Seafarers' Sleep on Board.	Inquiry	Seafarers, sleep quality, fatigue, mixed methods.	(16)
12	Szafran-Dobrowolska J, et al.	2023	The psychosocial burden and stress coping strategies among seafarers.	Int Marit Health	Seafarers, psychosocial burden, stress coping strategies.	(17)
13	Lazuk D, et al.	2025	Systematic review: psychomental reactions of survivors after fatal maritime disasters at sea.	Int Marit Health	Maritime disasters, survivors, psychomental reactions.	(18)
14	Stoll E, et al.	2020	Prevalence of alcohol consumption among seafarers and fishermen.	Int Marit Health	Seafarers, fishermen, alcohol consumption.	(19)
15	Jiang H, et al.	2018	Work Stress and depressive symptoms in fishermen with a smoking habit: a mediator role of nicotine dependence and a possible moderator role of expressive suppression and cognitive reappraisal.	Front Psychol	Fishermen, work stress, depression, smoking, emotional regulation.	(20)
16	Yadav OP, et al.	2021	Occupational noise exposure and health impacts among fish harvesters: a systematic review.	Int Marit Health	Fish harvesters, noise exposure, health impacts, systematic review.	(21)
17	Vukić L, et al.	2021	Seafarers' Perception and Attitudes toward Noise Emission on Board Ships.	Int J Environ Res Public Health	Seafarers, noise perception, attitudes.	(22)
18	Yadav OP, et al.	2023	Occupational noise exposure at sea: a socio-legal study on fish harvesters' perceptions in Newfoundland and Labrador, Canada.	Front Public Health	Fish harvesters, noise exposure, socio-legal study.	(23)
19	Remmen LN, et al.	2017	Fatigue and workload among Danish fishermen.	Int Marit Health	Danish fishermen, fatigue, workload.	(24)
20	Miñarro S, et al.	2022	Does catching more fish increase the subjective wellbeing of fishers? Insights from Bangladesh.	Ambio	Fishers, subjective wellbeing, catch quantity.	(25)
21	Jensen HJ and Oldenburg M	2021	Objective and subjective measures to assess stress among seafarers.	Int Marit Health	Seafarers, stress assessment, objective vs subjective measures.	(26)
22	Baygi F, et al.	2022	Psychosocial issues and sleep quality among seafarers: a mixed-methods study.	BMC Public Health	Seafarers, psychosocial issues, sleep quality, mixed methods.	(27)
23	Hayes-Mejia R and Stafström M	2024	Wellbeing and Happiness and their association with working conditions at sea: a cross-sectional study among the global workforce of seafarers.	Inquiry	Seafarers, wellbeing, happiness, working conditions.	(28)
24	Svetina M, et al.	2024	Factors impacting seafarers' mental health and career intentions.	Inquiry	Seafarers, mental health, career intentions.	(29)
25	Carrera-Arce M, et al.	2022	Healthy workplace onboard: insights gained from the COVID-19 impact on the mental health and wellbeing of seafarers.	Work	Seafarers, COVID-19, mental health, healthy workplace.	(30)
26	Nittari G, et al.	2022	Factors affecting the mental health of seafarers on board merchant ships: a systematic review.	Rev Environ Health	Seafarers, mental health factors, systematic review.	(31)
27	Sanz-Trepiana L, et al.	2024	Aggression, psychological violence, and sexual harassment among seafarers in France.	Int Marit Health	Seafarers, aggression, psychological violence, sexual harassment.	(32)
28	Chen Q, et al.	2020	Livelihood Vulnerability of Marine Fishermen to Multi-Stresses under the Vessel Buyback and Fishermen Transfer Programs in China: the case of Zhoushan City, Zhejiang Province.	Int J Environ Res Public Health	Marine fishermen, livelihood vulnerability, vessel buyback program.	(33)
29	Liang X and Yang Z.	2024	Subjective wellbeing of retired fishermen from the perspective of system-empowerment: an empirical study based on the Anhui section of the Yangtze river.	Chinese Fisheries Economics	Retired fishermen, subjective wellbeing, system empowerment (Chinese).	(34)
30	Gullot-Wright, et al.	2025	Social determinants of occupational injuries among US-based commercial fishermen: a systematic review.	Int J Equity Health	Commercial fishermen, occupational injuries, social determinants.	(35)
31	Abila SS and Acejo IL	2021	Mental health of Filipino seafarers and its implications for seafarers' education.	Int Marit Health	Filipino seafarers, mental health, education implications.	(36)
32	Chen S.	2022	A study on the intervention of case work in relieving seafarers' psychological fatigue.	Dalian Maritime University	Seafarers, psychological fatigue, case work intervention (Chinese, Thesis).	(37)
33	Le Gac JM and Texier S.	2022	Training in the detection of psychological distress on board ships through health simulation during the COVID-19 epidemic.	Int Marit Health	Psychological distress, training, health simulation, COVID-19.	(38)
34	Paranthatta S, et al.	2024	Effect of cyclic meditation on anxiety and sleep quality in sailors on merchant ships-A quasi-experimental study.	Front Public Health	Sailors, cyclic meditation, anxiety, sleep quality, intervention.	(39)
35	Leite MCF, et al.	2023	Social wellbeing, values, and identity among Caiçara small-scale fishers in southeastern Brazil.	Marit Stud	Small-scale fishers, social wellbeing, values, identity.	(40)
36	Xu Z, et al.	2023	Availability and access to livelihood capital assets for the development of sustainable livelihood strategies of fishermen: a case study of Manchar Lake, Pakistan.	Heliyon	Fishermen, livelihood capital, sustainable livelihood strategies.	(41)
37	Xiao J, et al.	2017	Association between social support and health-related quality of life among Chinese seafarers: a cross-sectional study.	PLoS ONE	Chinese seafarers, social support, health-related quality of life.	(42)
38	An J, et al.	2022	Empirical study on the relationship between vacation schedule and seafarers' fatigue in the chinese seafarer population.	Front Psychol	Chinese seafarers, vacation schedule, fatigue	(43)
39	Putri RD, et al.	2024	Improving small-scale fishermen's subjective wellbeing in Indonesia: does internet use play a role?	Heliyon	Small-scale fishermen, subjective wellbeing, internet use.	(44)
40	Pauksztat B, et al.	2022	The impact of the COVID-19 pandemic on seafarers' mental health and chronic fatigue: beneficial effects of onboard peer support, external support, and Internet access.	Mar Policy	Seafarers, COVID-19, mental health, chronic fatigue, peer support, internet.	(45)
41	Abila S, et al.	2023	Empowering seafarers as agents of their mental health: the role of information and communication technology in seafarers' wellbeing.	Inquiry	Seafarers, mental health empowerment, ICT, wellbeing.	(46)

## 5 Conclusion

In this scoping review, we find that fishermen's mental health constitutes a field of inquiry where the evidence base is critically insufficient, even as the challenges it confronts are undeniably severe. We argue that the body of research on seafarer mental health offers an indispensable, though imperfect, lens—it helps illuminate the common challenges shared by these two maritime occupational groups. More critically, through this comparison, we seek to foreground the special predicaments that uniquely define the fishermen's experience: predicaments rooted not only in their close ties to livelihood and macro-policy shifts, but also in the deep and often invisible shaping force of traditional culture. From our perspective, a supportive system for fishermen's mental health is far from established; what is urgently needed is a more comprehensive, empathetic understanding of their lived needs. It is our contention that only such understanding can inform the design of targeted new policies capable of safeguarding their physical and mental wellbeing. We therefore insist that future research and intervention design must be carefully, even meticulously, tailored to the unique—often informal, community-based, and culturally embedded—reality of fishermen's lives. We conclude by reiterating, with emphasis, the caution raised in our discussion: while seafarer research provides an invaluable reference point, the fundamental structural differences in occupational and social contexts between these two groups mean that solutions cannot be directly transplanted.
